# Plasma Levels of Apoliporptoein A1 in Malaria-Exposed Primigravidae Are Associated with Severe Anemia

**DOI:** 10.1371/journal.pone.0008822

**Published:** 2010-01-21

**Authors:** David C. Simpson, Edward Kabyemela, Atis Muehlenbachs, Yuko Ogata, Theonest K. Mutabingwa, Patrick E. Duffy, Michal Fried

**Affiliations:** 1 Seattle Biomedical Research Institute, Seattle, Washington, United States of America; 2 MOMS Project, Seattle Biomedical Research Institute, Seattle, Washington, United States of America; 3 Muheza Designated District Hospital, Muheza, Tanzania; 4 National Institute for Medical Research, Dar es Salaam, Tanzania; 5 University of Washington, Seattle, Washington, United States of America; Université Pierre et Marie Curie, France

## Abstract

**Background:**

*Plasmodium falciparum* placental malaria (PM) contributes to 10,000 maternal deaths due to severe anemia (SA) each year in Africa, primarily among primigravid women who are most susceptible. Increased levels of proinflammatory cytokines like TNF-α are associated with maternal anemia in first time mothers but not in other women. Here we aimed to identify additional changes in the plasma proteome associated with pregnancy malaria that may contribute to the development of malaria-related maternal anemia.

**Principal Findings:**

A semi-quantitative mass spectrometry approach was used to compare the relative abundance of plasma proteins in anemic versus non-anemic women with PM. Levels of 24 proteins differed significantly between anemic and non-anemic primigravidae, including several lipid metabolism proteins and molecular transport proteins involved in the acute phase response signaling network. These differences were not observed in multigravid women who enjoy specific immunity that protect them from PM. In a confirmatory study of a larger cohort of primigravid women, levels of the lipid metabolism protein Apolipoprotein (Apo)-AI were significantly lower in PM+ women with SA.

**Conclusions:**

Apo-AI levels are significantly lower in severely anemic primigravidae with PM, and ApoA1 levels positively correlate with hemoglobin levels in primigravid but not multigravid women. Apo-AI is known to have anti-inflammatory effects, and thus Apo-AI reductions may contribute to the inflammatory processes that result in SA.

## Introduction

Severe anemia (SA) is a common consequence of *P. falciparum* infection, and is a major cause of childhood and maternal mortality in Africa [1], [2], [3]. During pregnancy, *P. falciparum*-infected erythrocytes (IE) bind chondroitin sulfate A (CSA) [4] and accumulate in the placenta. Disease due to placental malaria (PM) is strongly associated with the macrophage-rich inflammatory infiltrates that sometimes appear in the intervillous spaces of the placenta. Women are most susceptible to infection, inflammation and disease during first pregnancy (reviewed in [5]), and become resistant over successive pregnancies as they acquire antibodies that block IE binding to CSA [6].

Pregnancy is an event of immunologic tolerance, whereby a woman accepts the implantation of the fetal allograft in her uterus. Normal placentas display a bias toward type 2 cytokines [7], but type 1 cytokine levels increase during PM [8], [9]. TNF-α levels increase in primigravid (first pregnancy) and multigravid (multiple prior pregnancies) women during PM, but are associated with SA in primigravid women only, suggesting that unknown factors in addition to TNF-α may be involved in the pathogenesis of SA [8].

The completion of several genome sequencing projects and the development of new proteomics tools provide an opportunity to study global changes associated with specific disease. In general, disease proteome studies that examine global changes in protein expression have sought to identify disease biomarkers, new therapeutic targets, and to understand host-pathogen interactions. The advantage of this approach is that it is not limited to the measurement of predicted factors, but instead assesses global changes associated with disease. This approach has been mainly used in studies of chronic diseases like cancer and vascular disease.

Here we report the first analysis of the plasma proteome during malaria, using samples collected from infected pregnant women with and without SA. Primigravid but not multigravid women had significantly altered levels of proteins involved in lipid metabolism and inflammatory immune responses. Similar changes have previously been reported in studies of other severe inflammatory diseases.

## Materials and Methods

### Subjects

Samples used for these studies were collected from women who delivered at New Nyanza Provincial General Hospital, Kisumu, Kenya, or at the Muheza Designated District Hospital, Muheza, Tanzania. Parturients 18 years and older were invited to participate in the study, and gave signed informed consent after receiving a study explanation form and oral explanation from a nurse in their native language. Women with chronic or debilitating disease were excluded from the study. These studies were approved by the International Clinical Studies Review Committee of the Division of Microbiology and Infectious Diseases at the US National Institutes of Health, and ethical clearance was obtained from the Institutional Review Board of Seattle Biomedical Research Institute, the National Institute for Medical Research in Tanzania and the Kenya Medical Research Institute.

### Clinical Data

Clinical information and medical history were recorded by study nurses using a standard questionnaire. Peripheral blood samples were collected by venipuncture immediately after delivery in citrate phosphate dextrose. Malaria infection status at the time of delivery was determined by microscopic examination of Giemsa-stained placental and peripheral blood smears. Hemoglobin concentrations were determined using Cell-Dyne 1200 hematology analyzer (Abott Diagnostics, North Chicago, IL, USA). SA was defined as hemoglobin level of <7 g/dl in pregnant women. Plasma was separated by centrifugation, and aliquots were stored at −80°C.

### Sample Selection

For global plasma proteome analysis, samples from malaria-infected primigravidae and multigravidae with and without SA were selected from two cohorts of women (Kenya and Tanzania). For confirmatory studies by ELISA all available samples from malaria-infected and uninfected primigravid and multigravid women with SA were analyzed. Samples from the same groups of women without SA were randomly selected for ELISA analysis.

### Preparation of Plasma Samples for Proteomics Studies

Six high-abundance plasma proteins were depleted using the Agilent (Palo Alto, CA, USA) Multiple Affinity Removal System Human 6 LC Column immunoaffinity-based depletion system according to the manufacturer's instructions. The proteins removed were albumin, IgG, IgA, transferrin, haptoglobin, and antitrypsin. The flow-through fraction containing unbound proteins was collected and concentrated using a 5 kDa molecular weight cutoff spin concentrator (Agilent). Samples were denatured and reduced by adding urea to 8 M, dithiothreitol to 10 mM, and then incubating for 1 h at 37°C. Cysteines were alkylated by adding iodoacetamide to 40 mM and then incubating for 1 h at 37°C. The reaction was quenched by adding dithiothreitol to a final concentration of 50 mM and then incubating for 1 h at 37°C. After a ten-fold dilution, the reduced and alkylated samples were digested overnight at 37°C using sequencing grade modified trypsin (Promega, Madison, WI, USA) at a 1∶50 enzyme:protein (w/w) ratio. The resulting peptide mixtures were de-salted using DSC-18 solid-phase extraction cartridges (Supelco, Bellefonte, PA, USA). 500 µg of peptide samples were acidified and fractionated by strong cation exchange (SCX) chromatography on a 2.1×200 mm Polysulfoethyl A column (PolyLC, Columbia, MD, USA). A gradient from 75% 10 mM formic acid (aq)/25% acetonitrile (A) to 75% 500 mM ammonium formate (aq)/25% acetonitrile (B) over 45 min was used to achieve separation. Fractions containing peptides underwent a final de-salting step using Vydac C18 Silica solid-phase extraction spin columns (The Nest Group, Southborough, MA, USA). Peptide concentrations were determined using a BCA assay kit (Pierce, Rockford, IL, USA).

### LC-MS/MS Analysis

LC-MS/MS was performed using an LTQ linear ion trap mass spectrometer (ThermoFinnigan, San Jose, CA, USA). The LC system consisted of a fused-silica nanospray needle packed in-house with C18 reversed-phase material. 2 µg of total peptide at 0.1 µg/µL were loaded onto the reversed phase column using a two-mobile-phase solvent system consisting of 0.4% acetic acid in water (A) and 0.4% acetic acid in acetonitrile (B). The mass spectrometer operated in a data-dependent MS/MS mode over the *m/z* range of 400–1400. For each cycle, the five most abundant ions from each MS scan were selected for MS/MS analysis using 20% normalized collision energy. Dynamic exclusion was used to exclude ions that had been detected twice in a 30 sec window for 45 seconds.

### Data Analysis

Raw MS/MS data were submitted to BioWorks 3.0 (ThermoElectron, San Jose, CA, USA) and searched using the Sequest algorithm against a combined database of human and *P. falciparum* 3D7 protein sequences. The database was produced by downloading all human and *P. falciparum* 3D7 sequences available from NCBI GenBank on May 25, 2006 (based on GenBank release 153). Sequest output was analyzed and validated by PeptideProphet [10]. Peptides with a probability score of ≥0.9 were accepted. The lists of detected peptides were converted to a list of corresponding unique genes using the cross-reference table provided by NCBI (ftp://ftp.ncbi.nlm.nih.gov/gene/DATA/gene2accession.gz). To estimate the false discovery rate, a reversed database approach was used [11]. The false discovery rate was estimated to be <1% at the mass spectrum level and <2% at the peptide level.

### Proteins Networks and Functional Analysis

Differential expression of plasma proteins was estimated by comparing spectrum counts [12], [13], and significant differences determined by Mann-Whitney *U* test using Statview software (SAS Inc, Cary, NC, USA).

Protein interaction networks and functions of differentially expressed proteins were analyzed using Ingenuity Pathways Analysis (Ingenuity Systems®, www.ingenuity.com). This program identifies networks of interacting proteins that are significantly enriched in the experimental dataset of differentially expressed proteins. Functional analysis of the dataset for molecular and cellular function, physiological system development and function, and disease and disorder, identifies predominant functions associated with the network. Statistical significance is calculated by comparing the number of proteins participating in a given function with the total number of the proteins known to have this function in the Ingenuity knowledge base.

### ELISA Assays

Apolipoprotein AI (Apo-AI) and Apolipoprotein B (Apo-B) concentrations were measured by a sandwich ELISA method using kits (AlerChek Inc, Portland, ME, USA) according to the manufacturer's instructions.

## Results

### Global Plasma Proteome by LC-MS/MS

Our goal in this study was to identify changes in the plasma proteome of *P. falciparum* infected pregnant women that are associated with SA. Plasma samples from 12 malaria-infected primigravidae (seven with SA and five without SA) and 12 samples from malaria-infected multigravidae (six with SA and six without SA) were analyzed. Mean placental parasite densities and mean hemoglobin levels were similar in the primigravid and multigravid women included in this study (Table 1).

**Table 1:** Characteristics of the study population in the mass spectrometry based proteomics study.

	Primigravidae *Mean (SD)*	Multigravidae *Mean (SD)*	*P value*
Maternal age (yr)	19.4 (1.6)	26.5 (4.5)	<0.0001
Placenta parasitemia (%)	2.2 (2.4)	1.04 (1.4)	NS*
Hemoglobin (g/dl)	6.6 (1.96)	8 (2.9)	NS*

* NS not significant.

In areas of high malaria incidence, anemia of primigravid women is commonly related to malaria while anemia of multigravid women is more likely to result from nutritional deficiencies [14]. The inclusion of plasma samples from malaria-infected primigravid and multigravid women enabled us to identify plasma proteome changes specific to malaria-associated SA (*ie*, found in primigravid but not multigravid women).

Global proteomic analysis yielded a dataset which corresponded to 2072 distinct proteins. This set was further filtered to include only those proteins that had at least two peptides detected in 25% or more of the samples in at least one of the groups. The final dataset included 156 proteins.

To identify global changes associated with SA, we quantified protein levels in each sample according to the number of MS/MS spectra corresponding to each of the proteins (Table S1). Using spectrum count as a semi-quantitative approach is based on the model that the number of observed spectra is directly related to the relative abundance of the protein [12], [15]. This model was previously validated by correlating protein concentrations and spectrum counts in samples spiked with known amounts of markers [12], [16]. Spectrum count has been used in comparative analyses of the urine proteome and to determine proteome changes in different cells under varying growth conditions [13], [17], [18]. Although this approach is semi-quantitative, we applied this tool to identify potential differences among samples with different clinical presentations that can be further evaluated using accurate quantitative methods like ELISA.

Based on relative protein abundance, 20 proteins were downregulated and 4 proteins were upregulated in plasma samples from primigravidae with SA versus those without SA (Table 2). GO (gene ontology) analysis for biological function of these proteins identified enrichment of proteins that have a role as transporters (Table 2). These differences were not observed in the comparisons of plasma samples from multigravid women with and without SA, except for ITIH4 for which the spectrum count was lower in women with SA (*p* = .05). Among multigravid women with SA, 13 other proteins were differentially expressed compared to multigravid women without anemia (Table S1).

**Table 2:** Differentially expressed proteins in plasma samples of primigravid women with SA versus those with no SA.

Accession number	Gene symbol	Ratio SA/No SA	*P value* (Mann-Whitney U test)	Protein type
NP_001624.1	AMBP	0.71	0.034	transporter
NP_000030.1	APOA1	0.64	0.042	transporter
NP_001634.1	APOA2	0.30	0.0045	transporter
NP_000375.1	APOB	0.21	0.0145	transporter
NP_001636.1	APOC1	0.45	0.041	transporter
NP_000474.2	APOC2	0.38	0.034	transporter
NP_000031.1	APOC3	0.23	0.0045	transporter
NP_001638.1	APOD	0.65	0.025	transporter
NP_000704.1	BLVRB	5.60	0.0088	enzyme
NP_000706.1	C4BPA	0.42	0.0043	other
NP_000177.2	CFH	0.72	0.041	other
NP_001349.2	DHX15	0.25	0.0067	enzyme
NP_000496.1	F12	1.62	0.05	peptidase
NP_000499.1	FGA	0.31	0.0045	other
NP_005132.2	FGB	0.31	0.0045	other
NP_000500.2	FGG	0.37	0.0118	other
NP_002017.1	FN1	0.22	0.0045	enzyme
NP_066275.3	HPR	5.51	0.0081	peptidase
NP_002209.2	ITIH4	0.57	0.0045	other
NP_000883.2	KLKB1	0.55	0.01	peptidase
NP_005568.1	LPA	0.02	0.0015	other
NP_005800.3	PRDX2	3.20	0.022	enzyme
NP_006503.1	SAA4	0.58	0.049	transporter
NP_006513.1	WNT6	0.40	0.025	other

### Interaction Networks and Canonical Pathway Analysis

To infer functional implications from the plasma proteome changes associated with SA during PM, we examined biological interaction pathways for the 24 differentially expressed proteins, using Ingenuity Pathways Analysis (IPA) software. Unlike traditional statistical analysis, pathway analysis provides overall information related to changes observed under different physiological conditions. Changes in the expression level of multiple proteins within a pathway increase the confidence that the differential expression of a single protein using traditional statistical methods is likely to be associated with the studied condition [19].

**Figure 1:**
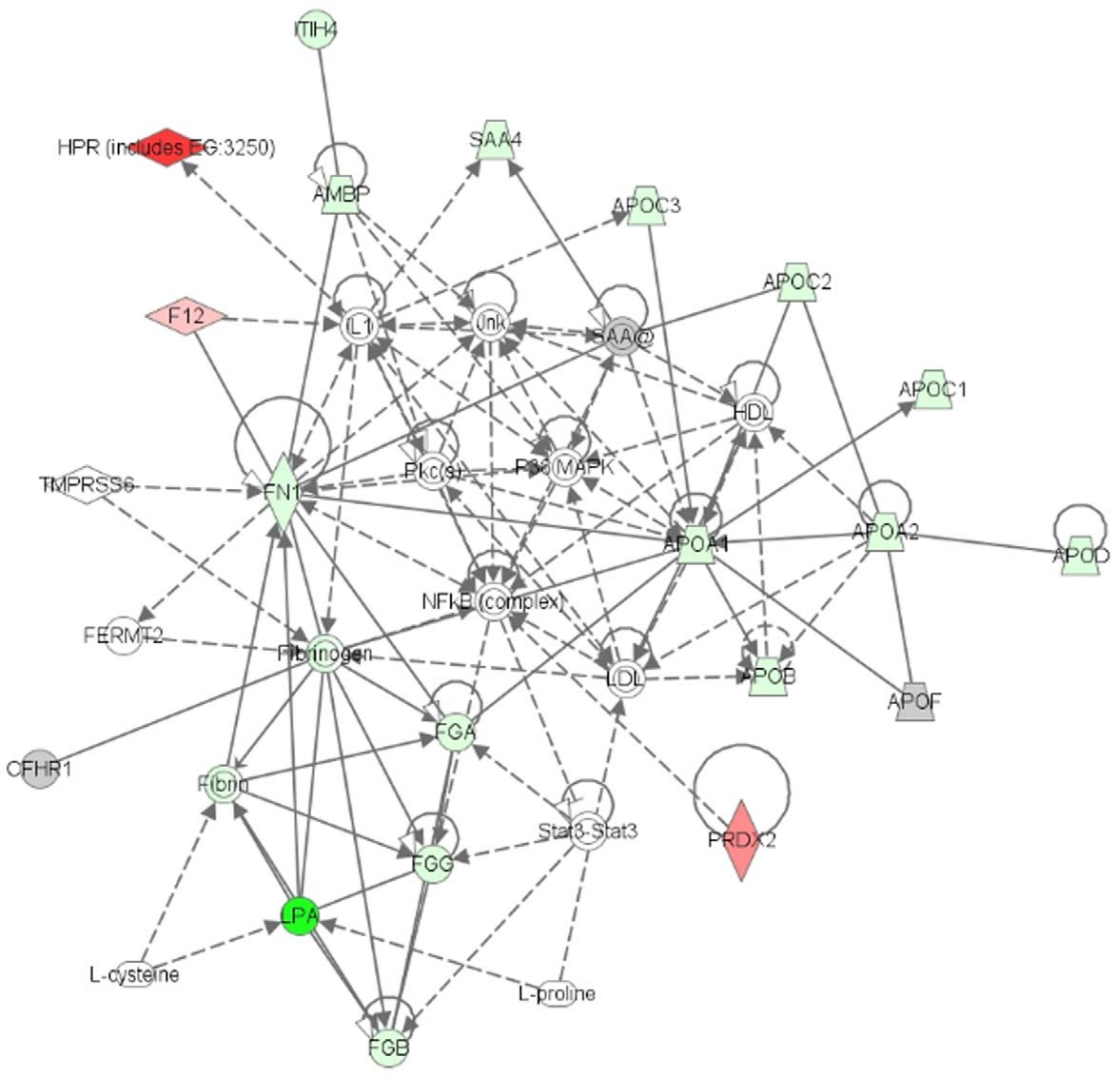
Network of differentially expressed plasma proteins of primigravid women with SA vesus primigravid women without SA. 24 proteins were eligible for network analysis by Ingenuity Pathways Analysis. The top-scoring network, illustrated here, included 18 focus proteins. Green and red symbols represent proteins that were down- and up-regulated during SA respectively. Gray symbols represent proteins identified in the study for which the difference in expression level did not achieve statistical significance. Direct and indirect interactions are represented by the solid and broken lines respectively.

Of the 24 differentially expressed proteins, all were eligible for network analysis based on information available for each gene in the IPA Knowledge Base. Two interaction networks were identified for the proteins associated with SA (Table 3). The highest scoring network (Figure 1) incorporated 18 out of the 24 differentially expressed proteins (focus molecules) indicated in Table 2, and the second network incorporated 5 focus molecules.

**Table 3:** Interaction networks defined by Ingenuity Pathways Analysis.

ID	Molecules in Network	Score	Focus Molecules in Network (in bold)	Top Functions
1	**AMBP**, **APOA1**, **APOA2**, **APOB**, **APOC1**, **APOC2**, **APOC3**, **APOD**, APOF, CFHR1, **F12**, FERMT2, **FGA**, **FGB**, **FGG**, Fibrin, Fibrinogen, **FN1**, HDL, **HPR** (includes EG:3250), IL1, **ITIH4**, Jnk, L-cysteine, L-proline, LDL, **LPA**, NFkB (complex), P38 MAPK, PKc(s), **PRDX2**, **SAA4**, SAA@, Stat3-Stat3, TMPRSS6	47	18	Lipid metabolism Small molecule biochemistry Cardiovascular disease
2	AIFI, ANPEP, APCS, ATP6V1B2, **BLVRB**, **C4BPA**, C4BPB, CAT, CDKN3, **CFH**, chondroitin sulfate A, CKS2, CMA1, CP, CTSK, **DHX15**, EMR1, heparin, IGFBP6, IL6, KITLG, **KLKB1**, LBP, LDLR, LIPC, PRG2 (includes EG:5553), PROS1, RARRES2, retinoic acid, SAA2, SERPINA5, SMAD5, TPSB2, UBE2C	10	5	Antigen presentation Cell-mediated immune response Humoral immune response

Four significant functions were associated with these networks: lipid metabolism (11 proteins, *p* = 6.98×10^−8^), small molecule biochemistry (16 proteins, *p* = 6.98×10^−8^), molecular transport (14 proteins, *p* = 1.2×10^−7^), and protein synthesis (7 proteins, *p* = 1.68×10^−7^). By IPA analysis, plasma proteome changes in malaria-infected primigravidae with SA resulted in alterations of five canonical pathways, most significantly the acute phase response signaling pathway. The plasma proteins associated with SA were represented in this pathway with 11 proteins out of the 178 proteins assigned to the pathway (*p* = 2.77×10^−16^).

### ELISA Studies

**Figure 2:**
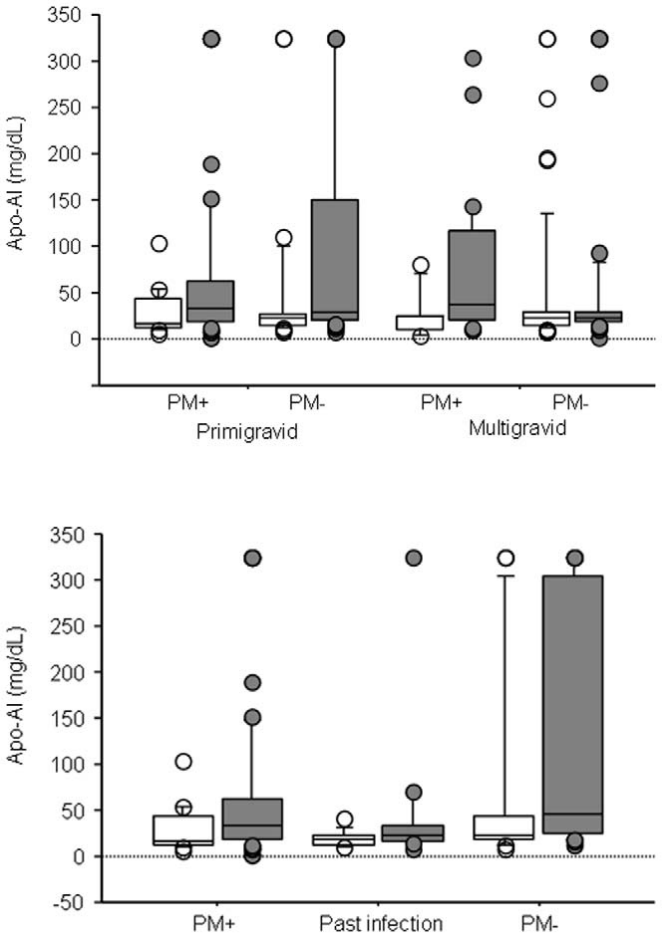
Comparison of peripheral plasma levels of Apo-A1 between malaria-infected and uninfected women with and without severe anemia. (A) Apo-AI levels in severely anemic (open boxes) and non-anemic (gray boxes) women stratified by gravidity and malaria infection status. Number of samples tested (severely anemic, non-anemic) and the significance of differences between anemic and non-anemic women (p value analyzed by Mann-Whitney U test): primigravid, PM+ (17, 58; *p* = 0.016); primigravid, PM- (27, 48; *p* = 0.015); multigravid, PM+ (7, 26; *p* = 0.11); multigravid, PM- (54, 58; *p* = 0.6). (B) Apo-AI levels in severely anemic (open boxes) and non-anemic (gray boxes) primigravid women stratified by 3 stages of infection: active infection (PM+), past infection, and no infection (PM-). Number of samples tested (severely anemic, non-anemic) and the significance of differences between anemic and non-anemic primigravid women (p value analyzed by Mann-Whitney U test): PM+ (17, 58; *p* = 0.016); past infection (11, 17; *p* = 0.18); PM- (16, 31; *p* = 0.06). The box plots include the median (middle line), interquartile ranges (box), and 10-90^th^ percentiles (whiskers).

By global plasma proteomic analysis, the relative abundance of seven apolipoproteins was lower among malaria-infected primigravidae with SA. Previous studies reported changes in HDL-cholesterol and LDL-cholesterol during malaria infection. We verified the changes observed in lipid metabolism proteins by measuring Apo-AI and Apo-B concentrations (representing HDL-cholesterol and LDL-cholesterol respectively) by ELISA in a larger group of samples collected from malaria-infected and uninfected primigravidae and multigravidae with and without SA (primigravidae n = 150; multigravidae n = 145). Plasma levels of Apo-AI were significantly lower in malaria-infected and uninfected primigravidae with SA versus those without SA (*p* = 0.016, *p* = 0.015 respectively). Differences in Apo-AI levels between women with and without SA were not observed among malaria-infected and uninfected multigravidae (Figure 2A). Among primigravidae, plasma Apo-AI levels positively correlated with hemoglobin levels in both malaria-infected and uninfected women (r = 0.285, *p* = 0.014 n = 75; r = 0.394, *p* = 0.0007, n = 75 respectively).

We further analyzed the relationship between SA, ApoA1 levels and past infection determined by histological examination of placenta tissue. Of the 75 samples classified as uninfected by placental blood smear, 28 had a history of past infection based on the presence of hemozoin deposits in fibrinoid. Apo-AI levels were higher in samples from uninfected women compared to those with active or past infection (*p* = 0.05, *p* = 0.0008 respectively). Among women with a history of PM by placental histology, Apo-AI levels were similar between those with and without SA (*p* = 0.18). Among uninfected primigravidae, Apo-AI levels were lower in those with SA but the difference did not achieve statistical significance (*p* = 0.06) (Figure 2B). Although Apo-AI levels were not significantly different between SA and non-anemic women, Apo-AI remained positively correlated with hemoglobin levels in samples from uninfected primigravidae with and without past infection (r = 0.398, *p* = 0.038, n = 28; r = 0.396, *p* = 0.007, n = 47). Defining placental infection history by placental histology could be limited by the time interval between infection and delivery. The correlation between ApoA1 and hemoglobin levels in primigravidae but not in multigravidae suggests that early PM events that may not be detected by placental histology could have a long term effect on anemia.

Previous studies suggested that pro-inflammatory cytokines like TNF-α can decrease levels of Apo-AI [20], [21]. Consistent with this, levels of TNF-α, IL-10 and IL-6 in placental blood (but not peripheral blood) were inversely correlated with peripheral Apo-AI levels in malaria-infected primigravidae (r = −0.385, *p* = 0.0017, n = 67; r = −0.484, *p* = 0.0002, n = 62; r = −0.353, *p* = 0.025, n = 50 respectively). Among uninfected primigravidae, levels of TNF-α and IL-10 in placental blood (but not peripheral blood) were inversely correlated with peripheral Apo-AI levels (r = −0.535, *p*<.0001, n = 70; r = −0.289, *p* = 0.02, n = 65). TNF-α remained inversely correlated with Apo-AI levels after excluding samples with evidence of past infection. Among malaria-infected multigravid women levels of TNF-α and IL-10 in placental blood (but not peripheral blood) were similarly inversely correlated with peripheral Apo-AI (r = −0.453, *p* = 0.011, n = 24; r = −0.392, *p* = 0.036, n = 22 respectively). A correlation between Apo-AI and cytokine levels was not observed in samples collected from uninfected multigravid women. During malaria infection, levels of TNF-α and IL-10 increase in both primigravid and multigravid women [8], [9], and could explain decreases in Apo-AI levels in these women.

**Figure 3:**
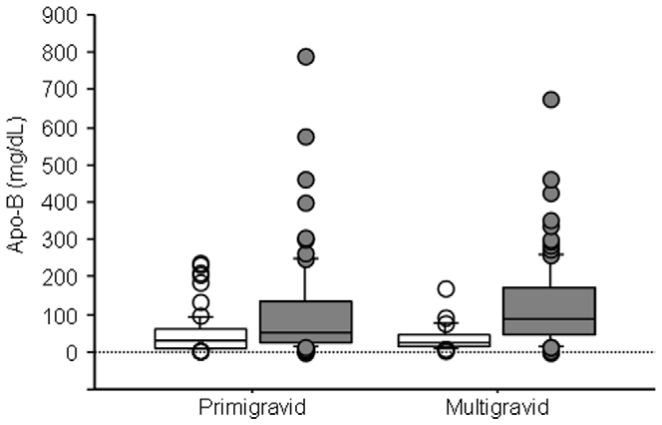
Comparison of peripheral plasma levels of Apo-B between malaria-infected primigravid and multigravid women. Apo-B in malaria-infected (open boxes) and uninfected (gray boxes) women stratified by gravidity. Numbers of samples tested (infected, uninfected) and the significance of the differences between malaria-infected and uninfected women (P value analyzed by Mann-Whitney U test): primigravid (75, 75; *p* = 0.0008; multigravid (33, 112; *p* = <.0001). The box plots include the median (middle line), interquartile ranges (box), and 10–90^th^ percentiles (whiskers).

By MS/MS data, Apo-B was significantly less abundant among primigravidae with SA (Table 2). Plasma levels of Apo-B measured by ELISA were significantly lower in both primigravidae and multigravidae with PM compared to uninfected women (Figure 3). However, Apo-B levels were similar in women with SA compared to those without SA.

## Discussion

Global proteomic analysis is not limited to the examination of known set of factors, and therefore has been a transformative technology that facilitates the identification of novel disease pathways and markers. In this study we employed mass spectrometry-based proteomics to better understand pregnancy malaria pathogenesis by identifying biomarkers associated with inflammation and anemia during placental malaria. We found that SA in infected primigravidae is associated with a significant change in proteins involved in lipid metabolism. Several previous studies found that severe infections or chronic inflammatory diseases other than malaria will also modulate the expression of proteins involved in lipid metabolism. During severe sepsis or chronic inflammation, pro-inflammatory cytokine and triglyceride (TG) levels are increased, while HDL-cholesterol, Apo-AI, Apo-B and LPa levels are decreased [22], [23], [24], [25]. Among patients under intensive care for severe sepsis, low levels of Apo-AI are associated with exacerbations of inflammation and poor prognosis [26].

Changes in lipid parameters have also previously been described in malaria-infected individuals. In several hospital-based studies of mild and severe malaria cases, TG levels were increased and HDL-cholesterol levels were decreased [27], [28], [29], [30]. In a study of asymptomatic children harboring low parasite levels, malaria treatment was followed by increases in HDL-cholesterol, LDL-cholesterol and total cholesterol levels, and a decrease in TG concentrations [31]. However, these changes were not associated with improvements in hemoglobin levels or other clinical parameters.

We earlier showed that SA is associated with high TNF-α levels in primigravid but not in multigravid women with PM, suggesting a role for other factors in the pathogenesis of SA. Apo-AI is a candidate factor for such a role. Apo-AI binds T cells *in vitro* and blocks contact with monocytes, thereby playing an anti-inflammatory role by inhibiting TNF-α and IL-1β production by monocytes [32]. Levels of pro-inflammatory cytokines and lipids change during chronic inflammatory diseases like rheumatoid arthritis (RA) [33]. Apo-AI levels decrease in the plasma but increase in synovial fluid of patients with active rheumatoid arthritis [34]. Immunohistological studies have co-localized Apo-AI with T lymphocytes and macrophages in the inflamed synovium from patients with active arthritis but not patients with inactive arthritis, suggesting that Apo-AI may have a role in limiting inflammation [35]. In parallel, TNF-α inhibits the expression of Apo-AI transcription in a dose-dependent manner [20], [21] suggesting a counter-regulatory relationship between TNF-α and Apo-AI during inflammatory responses.

Cytokines have a major role in stimulating the production of acute phase proteins (APP) as part of the host immune response to infection (reviewed in [36]), but prolonged inflammatory immune responses may be harmful to the host. Like albumin, Apo-AI is classified as a negative APP. While the role of negative APP is unclear, reduced levels of negative APP with anti-inflammatory activity, such as Apo-AI and transthyretin, may promote pro-inflammatory responses [35], [36]. This may be useful during the acute response to infection, but a prolonged pro-inflammatory environment may be deleterious to the host, leading to poor outcomes like anemia and impaired growth [36].

Several studies have identified associations between inflammatory immune responses and disease severity during malaria infection. In particular, TNF-α has been found to have both protective and harmful effects. TNF-α promotes parasite killing and has a protective role in controlling parasite levels, but high TNF-α levels have been associated with severe malaria syndromes like severe anemia and cerebral malaria [8], [37], [38]. In this study, Apo-AI levels negatively correlate with TNF-α levels in all women. Among primigravid women but not multigravid women, Apo-AI levels are significantly lower during SA. Multigravid women have specific immunity to placental parasites and are able to rapidly clear the infection, unlike primigravid women who lack specific immunity and often experience a prolonged pro-inflammatory state during PM. Moreover, a previous study demonstrated that the recovery in plasma lipoprotein levels after *P. vivax* infection is a slow process, with levels returning to normal 12 months after treatment [30]. We propose that the longer duration of PM or more frequent infection in primigravid women results in longer exposure to a proinflammatory environment that could lead to excessive reductions in Apo-AI levels and increases in the risk of SA.

## Supporting Information

Table S1 (0.05 MB XLS)

## References

[1] Zucker JR, Lackritz EM, Ruebush TK, Hightower AW, Adungosi JE (1996). Childhood mortality during and after hospitalization in western Kenya: effect of malaria treatment regimens.. Am J Trop Med Hyg.

[2] Fleming AF (1989). Tropical obstetrics and gynaecology. 1. Anaemia in pregnancy in tropical Africa.. Trans R Soc Trop Med Hyg.

[3] Shulman CE, Marshall T, Dorman EK, Bulmer JN, Cutts F (2001). Malaria in pregnancy: adverse effects on haemoglobin levels and birthweight in primigravidae and multigravidae.. Trop Med Int Health.

[4] Fried M, Duffy PE (1996). Adherence of Plasmodium falciparum to chondroitin sulfate A in the human placenta.. Science.

[5] Duffy PE, Desowitz RS, Duffy PE, Fried M (2001). Pregnancy malaria throughout history: Dangerous labor.. Malaria in Pregnancy: Deadly Parasite, Susceptible host.

[6] Fried M, Nosten F, Brockman A, Brabin BJ, Duffy PE (1998). Maternal antibodies block malaria.. Nature.

[7] Robertson SA, Seamark RF, Guilbert LJ, Wegmann TG (1994). The role of cytokines in gestation.. Crit Rev Immunol.

[8] Fried M, Muga RO, Misore AO, Duffy PE (1998). Malaria elicits type 1 cytokines in the human placenta: IFN-gamma and TNF-alpha associated with pregnancy outcomes.. J Immunol.

[9] Kabyemela ER, Fried M, Kurtis JD, Mutabingwa TK, Duffy PE (2008). Fetal responses during placental malaria modify the risk of low birth weight.. Infect Immun.

[10] Keller A, Nesvizhskii AI, Kolker E, Aebersold R (2002). Empirical statistical model to estimate the accuracy of peptide identifications made by MS/MS and database search.. Anal Chem.

[11] Moore RE, Young MK, Lee TD (2002). Qscore: an algorithm for evaluating SEQUEST database search results.. J Am Soc Mass Spectrom.

[12] Liu H, Sadygov RG, Yates JR (2004). A model for random sampling and estimation of relative protein abundance in shotgun proteomics.. Anal Chem.

[13] Gao J, Opiteck GJ, Friedrichs MS, Dongre AR, Hefta SA (2003). Changes in the protein expression of yeast as a function of carbon source.. J Proteome Res.

[14] Kabyemela ER, Fried M, Kurtis JD, Mutabingwa TK, Duffy PE (2008). Decreased susceptibility to Plasmodium falciparum in pregnant women with iron deficiency.. J Infect Dis In press.

[15] Washburn MP, Wolters D, Yates JR (2001). Large-scale analysis of the yeast proteome by multidimensional protein identification technology.. Nat Biotechnol.

[16] Zhang B, VerBerkmoes NC, Langston MA, Uberbacher E, Hettich RL (2006). Detecting differential and correlated protein expression in label-free shotgun proteomics.. J Proteome Res.

[17] Pang JX, Ginanni N, Dongre AR, Hefta SA, Opitek GJ (2002). Biomarker discovery in urine by proteomics.. J Proteome Res.

[18] Old WM, Meyer-Arendt K, Aveline-Wolf L, Pierce KG, Mendoza A (2005). Comparison of label-free methods for quantifying human proteins by shotgun proteomics.. Mol Cell Proteomics.

[19] Gerszten RE, Accurso FJ, Bernard GR, Caprioli RM, Klee EW (2008). Challenges in Translating Plasma Proteomics from Bench to Bedside: Update from the NHLBI Clinical Proteomics Programs.. Am J Physiol Lung Cell Mol Physiol.

[20] Kim JS, Oh JS, Chang EA, Bae SY, Nam DH (2008). Alteration of platelet counts and lipid profiles after treatment of acute Plasmodium vivax.. Acta Trop.

[21] Haas MJ, Horani M, Mreyoud A, Plummer B, Wong NC (2003). Suppression of apolipoprotein AI gene expression in HepG2 cells by TNF alpha and IL-1beta.. Biochim Biophys Acta.

[22] Beers A, Haas MJ, Wong NC, Mooradian AD (2006). Inhibition of apolipoprotein AI gene expression by tumor necrosis factor alpha: roles for MEK/ERK and JNK signaling.. Biochemistry.

[23] Alvarez C, Ramos A (1986). Lipids, lipoproteins, and apoproteins in serum during infection.. Clin Chem.

[24] Chenaud C, Merlani PG, Roux-Lombard P, Burger D, Harbarth S (2004). Low apolipoprotein A-I level at intensive care unit admission and systemic inflammatory response syndrome exacerbation.. Crit Care Med.

[25] Khovidhunkit W, Kim MS, Memon RA, Shigenaga JK, Moser AH (2004). Effects of infection and inflammation on lipid and lipoprotein metabolism: mechanisms and consequences to the host.. J Lipid Res.

[26] Mooser V, Berger MM, Tappy L, Cayeux C, Marcovina SM (2000). Major reduction in plasma Lp(a) levels during sepsis and burns.. Arterioscler Thromb Vasc Biol.

[27] Chien JY, Jerng JS, Yu CJ, Yang PC (2005). Low serum level of high-density lipoprotein cholesterol is a poor prognostic factor for severe sepsis.. Crit Care Med.

[28] Djoumessi S (1989). Serum lipids and lipoproteins during malaria infection.. Pathol Biol (Paris).

[29] Parola P, Gazin P, Patella F, Badiaga S, Delmont J (2004). Hypertriglyceridemia as an indicator of the severity of falciparum malaria in returned travelers: a clinical retrospective study.. Parasitol Res.

[30] Nilsson-Ehle I, Nilsson-Ehle P (1990). Changes in plasma lipoproteins in acute malaria.. J Intern Med.

[31] Faucher JF, Ngou-Milama E, Missinou MA, Ngomo R, Kombila M (2002). The impact of malaria on common lipid parameters.. Parasitol Res.

[32] Hyka N, Dayer JM, Modoux C, Kohno T, Edwards CK (2001). Apolipoprotein A-I inhibits the production of interleukin-1beta and tumor necrosis factor-alpha by blocking contact-mediated activation of monocytes by T lymphocytes.. Blood.

[33] Burger D, Dayer JM (2002). The role of human T-lymphocyte-monocyte contact in inflammation and tissue destruction.. Arthritis Res.

[34] Burger D, Dayer JM (2002). High-density lipoprotein-associated apolipoprotein A-I: the missing link between infection and chronic inflammation?. Autoimmun Rev.

[35] Bresnihan B, Gogarty M, Fitzgerald O, Dayer JM, Burger D (2004). Apolipoprotein A-I infiltration in rheumatoid arthritis synovial tissue: a control mechanism of cytokine production?. Arthritis Res Ther.

[36] Gabay C, Kushner I (1999). Acute-phase proteins and other systemic responses to inflammation.. N Engl J Med.

[37] Clark IA, Hunt NH, Butcher GA, Cowden WB (1987). Inhibition of murine malaria (Plasmodium chabaudi) in vivo by recombinant interferon-gamma or tumor necrosis factor, and its enhancement by butylated hydroxyanisole.. J Immunol.

[38] Grau GE, Taylor TE, Molyneux ME, Wirima JJ, Vassalli P (1989). Tumor necrosis factor and disease severity in children with falciparum malaria.. N Engl J Med.

